# Association between seminal plasma neopterin and oxidative stress in male infertility: A case-control study

**Published:** 2018-02

**Authors:** Tayebeh Ghiasvand, Mohammad Taghi Goodarzi, Gholamreza Shafiee, Alireza Zamani, Jamshid Karimi, Marzieh Ghorbani, Iraj Amiri

**Affiliations:** 1 *Research Center for Molecular Medicine, Hamadan University of Medical Sciences, Hamadan Iran. *; 2 *Department of Clinical Biochemistry, School of Medicine, Hamadan University of Medical Sciences, Hamadan, Iran.*; 3 *Endometer and Endometriosis Centre, Fatemieh Hospital, Hamadan University of Medical Sciences, Hamadan, Iran. *

**Keywords:** Infertility, Neopterin, Oxygen radical absorbance capacity, DNA damage

## Abstract

**Background:**

Neopterin is a significant and sensitive marker in estimating the activity of cellular immune system. Oxidative stress plays a role in the etiology of male infertility. Increased reactive oxygen species is accompanied with increase in neopterin level. Hence neopterin may be involved in male infertility.

**Objective:**

The objective of this case-control study was to determine neopterin level in idiopathic infertile and normospermic men; furthermore, to identify its relationship with oxidative stress markers including total oxidant, malondialdehyde, sperm DNA fragmentation, and total antioxidant capacity of seminal plasma.

**Materials and Methods:**

Forty seven infertile and forty three normospermic males were selected according to WHO criteria. Their semen and blood samples were taken; subsequently, the levels of neopterin, total oxidant, total antioxidant, malondialdehyde, and sperm DNA fragmentation were measured.

**Results:**

The levels of neopterin, total oxidant, and malondialdehyde in seminal plasma of infertile males were significantly higher than those of normospermic group (p=0.038, 0.018, and 0.028, respectively). Furthermore, sperm DNA fragmentation in infertile men was higher than that of control group (p<0.001). Moreover, total antioxidant capacity of seminal plasma in infertile males was significantly lower than that of normospermic subjects (p=0.002). No significant difference was observed in serum neopterin, total oxidant, and malondialdehyde between the infertile and normospermic groups.

**Conclusion:**

The significant inverse correlation between seminal plasma neopterin and total antioxidant in the infertile males supports a possible role of neopterin in male infertility. Neopterin can be suggested as a marker in monitoring and diagnosis of idiopathic male infertility.

## Introduction

Neopterin [D-erythro-6-(1´2´3´-trihydroxypropyl)-pterin] is the product of guanosine triphosphate catabolism. Interferon gamma is secreted by T-helper 1 cells and causes the secretion of neopterin by monocytes/macrophages (1, 2). A close relationship is present between the neopterin released from macrophages and their capacity for producing reactive oxygen species (ROS) (3). 

Measurement of neopterin concentration in body fluids such as serum, urine, cerebrospinal fluid, etc. is indicative of the activity of immune system by T-herper 1 cells. Typically, increased concentration of neopterin is observed in viral infections, intracellular parasites, autoimmune diseases, malignant tumors, cancer, cardiovascular diseases, oxidative stress, etc. (2, 4, 5). Neopterin is regarded as a crucial diagnostic marker of the activity, function of cellular immune system, and can be associated with prognosis, degree of disease progression, and monitoring of treatment (6).

Peroxidase-positive leukocytes consist of polymorphonuclear and macrophages, which constitute 50-60% and 20-30% of the whole seminal leukocytes, respectively (7). Peroxidase-positive leukocytes are the main sources of producing high levels of ROS in seminal plasma and are widely provided by seminal vesicles and prostate. ROS production capacity in leukocytes is associated with leukocytes activity in inflammatory and immune responses. Activated leukocytes are capable of producing ROS with the rate of 100 times more than that produced by inactivated leukocytes (8, 9). Furthermore, sperm in physiological conditions produces a small amount of ROS. 

Low concentrations of ROS play a significant role in signal transduction mechanisms, capacity of sperm, acrosome reaction, sperm binding to the ovules and their fusion, and ultimately fertility (10, 11). Furthermore, prospective studies have indicated that men with higher levels of ROS in comparison with those with lower levels have seven times less chance of fertility (12). Sperm plasma membrane contains high levels of ROS attack on polyunsaturated fatty acids in sperm membrane, whose cytoplasm has lower concentrations of scavenger enzymes, initiates the reaction cascade of lipid peroxidation, which widely leads to deterioration and destruction of polyunsaturated fatty acids. The mentioned process leads to the reduction of motility, fluidity, function, and ability of fertility (13). The final product of lipid peroxidation is malondialdehyde (MDA) that its measurement is used for monitoring the degree of peroxidative damage in sperm (14). 

Maintaining sperm DNA integrity plays a very significant role in transmission of genetic information, formation of embryo, and its growth and development (15). DNA can be changed and modified by many chemical mutagens like ROS, ultraviolet radiation, X-ray, gamma-ray, and alkylating agents (16, 17). ROS can cause gene mutations such as point mutations and polymorphisms and thereby reduce the quality of semen. Sperm DNA damage causes base degradation, DNA fragmentation, cross-linking of proteins, and reduced fertility of oocytes (18). Antioxidant activities of semen superoxide dismutase and catalase have more remarkable roles in defense against ROS (19). 

Furthermore, a reduction in the amount and activity of antioxidants such as superoxide dismutase, catalase, etc. in semen is associated with increased oxidative stress and idiopathic infertility (20). The mentioned points result in increased lipid peroxidation and decreased sperm motility, viability, and function, which ultimately lead to infertility. Based on definition of WHO “Infertility is the inability of a sexually active, non-contracepting couple to achieve spontaneous pregnancy in one year” (21).

The present study aimed at comparing the neopterin level of idiopathic infertile and normospermic male groups as well as specifying its relationship with oxidative stress markers including total oxidant, MDA, sperm DNA fragmentation, and total antioxidant capacity of seminal plasma. 

## Materials and methods

This case-control study was conducted in Research Center for Molecular Medicine and Infertility Clinic of Hamadan University of Medical Sciences, Hamadan, Iran. 

**Subjects**

Two groups containing 47 idiopathic infertile and 43 normospermic male within the age range of 20-40 yr participated in this study. Idiopathic infertile individuals were selected and introduced by an urologist according to World Health Organization criteria (21). The inclusion criteria for case group were having 20-40 yr age and inability to achieve pregnancy, in spite of non-countercepting sexual activity during the last one year. The exclusion criteria for case group were: smoking and having identified infertility causes including varicocele, cryptorchidism, renal and urinary tract infections, trauma, and chemotherapy.

Control group was selected among the subjects referring to the infertility center due to their spouses’ infertility or vasectomy operation. The inclusion criteria was having normal sperm quality and 20-40 yr age. The exclusion criteria for control group were: smoking, receiving any form of medication, and having any systemic disease.

**Sample prearation and Processing**

Two to four days after the last sexual intercourse, semen samples were collected through self-stimulation. As white blood cells are capable of producing ROS (22), the samples containing white blood cells and immature sperm were excluded. Each semen sample was kept in an incubator at 37^o^C for 30 min to undergo liquefaction. Then, semen analysis was manually conducted based on World Health Organization standards and criteria (23). Furthermore, sperm analysis was done with the utilization of Computer-Aided Sperm Analysis system and application of video test sperm 2.1. 

The samples were classified into infertile and normospermic groups. To separate the semen plasma from the sperm, semen samples were centrifuged at 500 g for 10 min; simultaneously, peripheral blood samples were taken. To separate serum, the blood samples were centrifuged at 1500 g for 10 min. Serum and seminal plasma were maintained at -70^o^C until the time of testing. 

**Biochemical Analysis**

Measurement of the levels of semen and serum oxidant was performed using ferric-xylenol orange assay (FOX) method (24). The measurement was based on ion oxidation of ferrous iron to ferric iron ions in the presence of an oxidant in the acidic environment. Total oxidant concentration of the samples was calculated considering standard curve of hydrogen peroxide at concentrations of zero to 100 micromolar. To measure MDA, Yagi method was used (25). The fluorescence intensity of the reaction product was measured using flourimetry (Jasco FP-6200, Tokyo, Japan) in the excitation wavelength of 515 nm and emission wave length of 553 nm. Tetra-ethoxypropane was used to plot the standard curve.

Semen and serum total antioxidants were measured using colorimetric microplate method (kit made by Cayman Company, Item No. 709001, USA). Neopterin in seminal plasma and serum was measured in accordance with the instructions provided in the ELISA kit made by DRG Company (EIA-2949, USA). The seminal plasma samples were diluted ten-fold to be exposed to the range of standard concentrations. 

Sperm DNA fragmentation was examined using Terminal deoxynucleotidyl transferase-mediated deoxyuridine triphosphate (dUTP) nick-endlabeling (TUNEL) test sperm by the In Situ Cell Death Detection Kit (Roche, Cat. No.11684795910, Germany). The percentage of cells with DNA fragmentation was determined by fluorescence microscopy (Ziess, Germany), which has high diagnostic value as it directly identifies single- and double-chain DNA using enzymatic binding of a fluorescent substance called (dUTP) to free 3'OH end of DNA strands. dUTP connected to these fluorescent fractures is detectable by fluorescent or light microscopy employing another technique known as Fluorescence-Activated Cell Sorting (26).

**Ethical consideration**

The study protocol was approved by the Ethics Committee of Hamadan University of Medical Sciences (Code: P/16/35/9/2), and informed consent was obtained from all the participants involved in the study.

**Statistical analysis**

Statistical analyses (pearson's correlation and t-test) was carried out using the Statistical Package for the Social Sciences version 16.0. SPSS Inc. Chicago, Illinois, USA (SPSS). p<0.05 was considered as significant level of the obtained results.

## Results

The mean age of the individuals in normospermic and infertile groups were 32.4 and 34.6 yr respectively; which was not significantly different, indicating age matched groups (Table I). Analysis of the standard semen parameters including motility, morphology, sperm count, and viability of the two groups revealed that the level of all the mentioned parameters were significantly lower in infertile group compared with that of the control group (p<0.001). 

The levels of total oxidant and MDA in seminal plasma of infertile patients were significantly higher than those of control group. Seminal plasma MDA presented a significant correlation with seminal plasma total oxidant in the infertile group (r=0.239, p=0.024). Furthermore, both infertile and control groups revealed a significant and direct correlation of serum MDA and total oxidant (r=0.393, p<0.001, r=0.452, p=0.002 respectively). 

**Figure 1:**
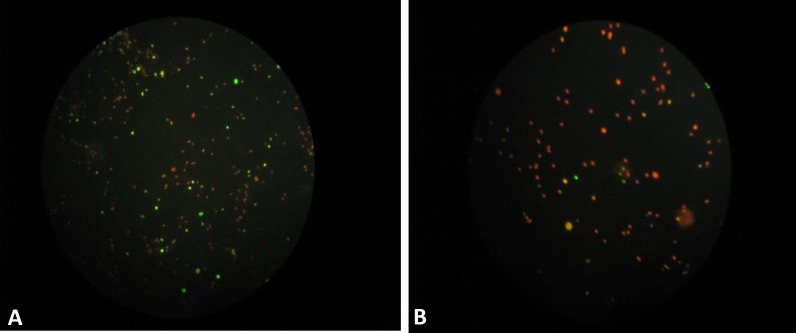
Tunel test results presenting DNA fragmentation, A) infertile and B) normal sperm with DNA fragmentation and sperm with intact DNA are shown as green and red points respectively (magnification×400

The level of total antioxidant capacity of seminal plasma in infertile men was significantly less than that of normospermic men. However, the serum levels of total oxidant and MDA in both groups were not significantly different. As presented in table I, seminal plasma neopterin level of infertile patients was significantly higher than that of the control participants (p=0.038). However, serum neopterin level in two groups showed no significant difference (p=0.193). Moreover, seminal plasma neopterin revealed a significant inverse correlation with total antioxidant in the infertile group (r=-0.395, p=0.009). Figure 1 presents an example of the TUNEL test results. Percentage of sperm cells with fragmented DNA was higher in infertile group comparing to control group (p<0.001, table I).

**Table I.** The results of semen analysis and measurement of neopterin, seminal plasma parameters, serum oxidative and antioxidative factors in infertile and normospermic groups

**Test parameter**		**Normospermic group (** **N= 43** **)**	**Infertile group (** **N= 47)**	**p-value**
Age (yr)		32.40 ± 3.9	34.6 ± 4.5	0.120
Semen parameters*				
	Volume (ml)	3.1 ± 0.83	2.7 ± 0.45	0.003
	Count (10 ^6^/ml)	75.12 ± 12.60	53.04 ± 28.41	<0.001
	Morphology (%)	31.51 ± 4.8	17.98 ± 12.06	<0.001
	Viability (%)	55.89 ± 6.40	30.48 ± 11.56	<0.001
	Motility (%)	38.00 ± 6.60	16.89 ± 8.88	<0.001
Seminal plasma total oxidant (μM)		4.85 ± 2.69	6.76 ± 4.52	0.018
Serum total oxidant (μM)		52.78 ± 22.40	59.90 ± 23.55	0.147
Seminal plasma total antioxidant (mM)		0.23 ± 0.11	0.14 ± 0.13	0.002
Seminal plasma neopterin (ng/ml)		289.7 ± 19.30	399. 4 ± 30.67	0.038
Serum neopterin (ng/ml)		5.27 ± 2.24	5.82 ± 2.72	0.193
Serum MDA (nmol/ml)		3.28 ± 1.67	3.76 ± 1.64	0.183
Seminal plasma MDA (nmol/ml)		0.69 ± 0.28	1.13 ± 1.28	0.028
DNA fragmentation (Tunel %)		13.56 ± 2.70	18.77 ± 2.32	<0.001

All data are expressed as mean±SD. t-test.

MDA: Malonedialdehyde

* The semen analysis is based on the: World Health Organization Manual for the examination and processing of human semen, in 5^th^ ed, 2010

## Discussion

The present study revealed that the level of seminal plasma neopterin was higher in infertile group compared with the control group and it showed a direct correlation with total oxidant and inverse correlation with total antioxidant capacity of seminal plasma. However, in the control group, the levels of neopterin and total oxidant were lower, and the level of total antioxidant capacity was higher. Both of the presented findings revealed a significant difference, which confirm the expected objectives. In addition, serum neopterin in both groups was in the normal range (<10 nmol) and did not present any significant differences. 

Moreover, measurement of seminal plasma neopterin compared with serum neopterin seems to be more plausible due to two reasons. First, serum neopterin is taken from the systematic blood stream, which is affected by all the organs and limbs; while, seminal plasma neopterin is merely influenced by male reproductive tissues. Second, serum neopterin test is an invasive test; while seminal plasma neopterin test can be considered as a non-invasive test.

Oxidative stress may be involved in the etiology of male infertility due to the production of excessive amounts of oxygen free radicals (20). Antioxidants act as free radical scavenger to protect sperm against ROS (27). Total antioxidant capacity of seminal plasma is reported to be lower in infertile men in comparison with fertile ones (28-30). Murr and colleagues declared that neopterin could be utilized as a marker for both cellular immune system activation and oxidative stress (31, 32). 

Furthermore, it has been specified that the increase of neopterin production is accompanied with increased production of oxygen free radicals and decreased concentration of antioxidants such as alpha-tocopherol. Hence, the amount of oxygen radicals produced by the activity of immune system can be estimated using neopterin concentration.

A close relationship is present between the neopterin released from macrophages and their capacity for producing ROS (3). Activated macrophages have two ways to damage the sperm: first, by producing high levels of reactive oxygen and nitrogen species that will lead to sperm membrane lipid peroxidation and oxidative DNA damage; secondly by releasing lysosomal enzymes and cytotoxic peptides (33, 34). Neopterin has been observed in various biological samples such as serum, cerebrospinal fluid, synovial fluid, ascitic fluid, urine, saliva, and pancreatic secretions (5, 35). Over recent years, a number of studies addressed the association of neopterin with a variety of diseases in humans. 

In a study conducted by Murr *et al* comparing the patients with coronary artery disease and healthy participants (36), it was revealed that the level of serum neopterin was high in the patients group, which was directly correlated with the level of ROS and inversely correlated with the level of antioxidants such as lycopene, beta-carotene, lutein, and ascorbic acid. Due to the existing relationship between neopterin and ROS production, neopterin concentrations in body fluids can be considered as indicative of the presence of oxidative stress and cellular immune system activity. In other words, the risk of cardiovascular disease is associated with the levels of neopterin, ROS, and serum antioxidant compounds (36). 

In 2008, Svoboda *et al* study showed that one of the neopterin derivatives called 8-hydroxy-2-deoxyguanosine (8-OHdG) increases with age (37). Furthermore, increased oxidant compounds such as 8-OHdG and neopterin can clearly be observed in the diseases that stimulate the immune response such as atherosclerosis, Huntington's and Alzheimer's diseases, and other autoimmune disorders (38). The study conducted by Wirleitner and coworkers revealed that neopterin can be used as a diagnostic marker for human immunodeficiency virus infection and acquired immunodeficiency syndrome (38). Berdowska and Melichar indicated that concentration of neopterin in colon, ovary, uterine cervix and kidney diseases, and breast cancers significantly increased; however, its level was significantly decreased in response to treatment (5, 2).

Tremellen and Tunc conducted the first and the single study addressing the presence of neopterin in semen liquid (39). Furthermore, they examined its relationship with sperm quality parameters such as number, morphology, motility, sperm oxidative stress markers, DNA fragmentation, and apoptosis (Annexin) in fertile and infertile men (39). They believed that as a common immunohistochemistry technique did not exist to measure the number of macrophages, neopterin measurement as indirect evidence and a non-invasive measurement tool provides the opportunity to measure the activity of macrophages in male reproductive tract (39). Their results indicated a significant relationship between neopterin and sperm quality parameters (39). 

Likewise, an increase of almost three times in the level of neopterin was observed in infertile men compared with fertile ones, and the prevalence of inflammation of the reproductive tract in infertile men was evidently higher than that of fertile ones. Our study also indicated similar results; however, it was a milder increase in neopterin (about 1.5 times). Moreover, they measured elastase, which is the activity index of neutrophil in seminal plasma; however, unlike neopterin, elastase presented no correlation with the quality sperm parameters (39). In recent study, the antioxidant enzymes were not measured; however, we found a lower level of seminal total antioxidants in infertile group. The other difference in the recent study with the above mentioned report was measurement of DNA fragmentation using TUNEL assay, which showed a higher percentage of sperm DNA fragmentation in infertile group. This indicates presence of higher level of reactive oxidant molecules in infertile group.

In the previous study conducted by the researchers of this article, serum levels of antioxidants lycopene, beta-carotene, and vitamin A in infertile and normospermic men were measured (40). Furthermore, the relationship of the mentioned factors with malondialdehyde and DNA fragmentation of sperm was examined. The obtained results suggested a significant decrease of the presented antioxidants in infertile group; furthermore, these antioxidants revealed significant and inverse correlation with MDA and sperm DNA fragmentation (40).

## Conclusion

Based on the findings discussed above, it is suggested to take the advantage of seminal plasma neopterin as a useful marker in the diagnosis and treatment of male infertility caused by cellular immunity and oxidative stress.
